# RAB31 in glioma‐derived endothelial cells promotes glioma cell invasion via extracellular vesicle‐mediated enrichment of MYO1C

**DOI:** 10.1002/2211-5463.13736

**Published:** 2023-11-20

**Authors:** Jinghao Suo, Yuxin Wang, Lin Wang, Bojun Qiu, Zhixing Wang, An Yan, Boqin Qiang, Wei Han, Xiaozhong Peng

**Affiliations:** ^1^ Department of Molecular Biology and Biochemistry, Institute of Basic Medical Sciences, Medical Primate Research Center, Neuroscience Center, Chinese Academy of Medical Sciences, School of Basic Medicine Peking Union Medical College Beijing China; ^2^ State Key Laboratory of Common Mechanism Research for Major Diseases Beijing China; ^3^ State Key Laboratory of Respiratory Health and Multimorbidity Beijing China; ^4^ National Human Diseases Animal Model Resource Center, Beijing Engineering Research Center for Experimental Animal Models of Human Critical Diseases，Institute of Laboratory Animal Science Chinese Academy of Medical Sciences & Peking Union Medical College Beijing China

**Keywords:** endothelial cells, extracellular vesicles, glioma, invasion, MYO1C, RAB31

## Abstract

Extracellular vesicles (EV), important messengers in intercellular communication, can load and transport various bioactive components and participate in different biological processes. We previously isolated glioma human endothelial cells (GhECs) and found that GhECs, rather than normal human brain endothelial cells (NhECs), exhibit specific enrichment of MYO1C into EVs and promote the migration of glioma cells. In this study, we explored the mechanism by which MYO1C is secreted into EVs. We report that such secretion is dependent on RAB31, RAB27B, and FAS. When expression of RAB31 increases, MYO1C is enriched in secretory EVs. Finally, we identified an EV export mechanism for MYO1C that promotes glioma cell invasion and is dependent on RAB31 in GhECs. In summary, our data indicate that the knockdown of RAB31 can reduce enrichment of MYO1C in extracellular vesicles, thereby attenuating the promotion of glioma cell invasion by GhEC‐EVs.

## Graphical abstract

Glioma human endothelial cells (GhECs) (unlike normal human brain endothelial cells (NhECs)), exhibit specific enrichment of MYO1C into EVs. Here, we identified an EV export mechanism for MYO1C that promotes glioma cell invasion and is dependent on RAB31 in GhECs. Knockdown of RAB31 reduces enrichment of MYO1C in extracellular vesicles, thereby attenuating promotion of glioma cell invasion by GhEC‐EVs.

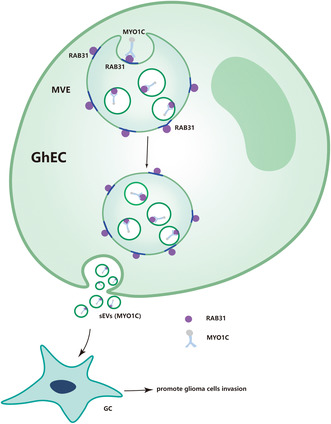

**Abbreviations**
EV: extracellular vesicles
GC: glioma cells
GhEC: glioma human endothelial cells
GSC: glioma stem cells
NhEC: normal human brain endothelial cells

Glioma is a highly vascularized tumor, and its perivascular microenvironment is crucial for the maintenance of the glioma cells (GCs) [1, 2]. On the one hand, the microenvironment promotes tumor‐related angiogenesis; on the other hand, the anoxic microenvironment aggravated by an abnormal vascular system promotes the invasion and progression of the tumor cells [3, 4]. Tumor endothelial cells also show abnormal biological phenotypes, such as reduced size, irregular morphology, and increased pinocytosis, and express specific molecules different from normal endothelial cells; these heterogeneous molecules can act as the key targets for tumor vascular therapy [5, 6, 7].

Extracellular vesicles (EV) are important carriers for intercellular communication and play an important role in tumor microenvironment [8]. Extracellular vesicles transport various cargo molecules and regulate metabolic activities of target cells through different mechanisms; therefore, they are widely involved in various physiological and pathological processes [9, 10]. The regulation of EV in target cells depends on their composition. Studies have shown that the composition of EV is not exactly the same as that of donor cells, suggesting the existence of specific cargo sorting and secretion mechanisms in the cell [11]. Extracellular vesicles can be divided into large extracellular vesicles (lEV) and small extracellular vesicles (sEV), according to their morphology and size. The lEV is mainly derived from budding of the cell membrane, whereas sEV is mainly derived from the endosomal membrane system [12]. During the development of endosome into multivesicular endosome (MVE), the endosomal membranes bud inward and selectively load cargo molecules, thus forming intraluminal vesicles (ILVs) [13, 14]. Subsequently, the fusion of MVE with the lysosomal membrane leads to the degradation of ILVs, while fusion with the cell membrane leads to the release of ILVs into the extracellular space, forming sEV.

We previously demonstrated that glioma human endothelial cell (GhEC)‐EV, rather than normal human brain endothelial cell (NhEC)‐EV, promote glioma cell migration, and MYO1C in GhEC‐EVs was significantly higher than that in NhEC‐EVs [15]. MYO1C is a member of the myosin‐I family [16] and is involved in many cellular processes related to membrane transport and morphology [17, 18]. MYO1C is usually expressed in the cytoplasm, and studies have shown that phosphorylated MYO1C can be involved in the transport of GLUT4 vesicles in adipocytes [19]. Studies on primary human endothelial cells have shown that MYO1C mediates VEGF‐induced recruitment and localisation of VEGFR2 to the cell membrane, thereby playing a role in the transmission of angiogenic signals [20]. In addition, the deficiency of functional MYO1C perturbs the cellular distribution of lipid rafts, leading to the accumulation of cholesterol‐rich membranes in the perinuclear circulation compartment, resulting in an enlarged lysosomal membrane [21]. However, the protein loading and secretion pathways of MYO1C have not been investigated. This study aimed to investigate the specific enrichment mechanism of MYO1C in GhEC‐EVs and provide a new theoretical basis for glioma treatment strategies targeting blood vessels. We found that MYO1C can promote GCs invasion and that its enrichment in GhEC‐EVs depends on RAB31.

## Materials and methods

### Cell culture

The Cell Resource Center, Peking Union Medical College (PCRC) provided the human umbilical vein endothelial cells (HUVECs) and human embryonic kidney 293T cells. Normal human brain endothelial cells (NhECs) were obtained from Cell Systems (Seattle, WA, USA). Glioma human endothelial cells (GhECs) were isolated in our laboratory according to the cell separation and purification method previously reported [22]. Endothelial cells (ECs) were grown in EBM‐2 medium (Lonza, Basel, Switzerland) supplemented with 2% fetal bovine serum (FBS), 0.1% hydrocortisone, 0.1% LR3IGF‐I, 0.4% hFGF‐b, 0.1% VEGF, 0.1% ascorbic acid, 0.1% GA‐1000, 0.1% hEGF, 0.1% heparin, 100 U·mL^−1^ penicillin, and 0.1 g·mL^−1^ streptomycin (Lonza). 293T cells was maintained in Dulbecco's modified Eagle medium supplemented with 10% FBS, 1 mm sodium pyruvate, penicillin (100 U·mL^−1^), and streptomycin (0.1 g·mL^−1^). The human glioma cell line U87MG was purchased from the American Type Culture Collection (ATCC) and maintained in modified Eagle's medium (MEM) supplemented with 10% FBS, 1 mm sodium pyruvate, 1% (v/v) nonessential amino acids (NEAA), penicillin (100 U·mL^−1^), and streptomycin (0.1 g·mL^−1^). Glioma stem cell lines GSC2 were obtained and cultured as previously reported [23]. All kind of cells were grown in a humidified incubator with 5% CO_2_ at 37 °C.

### Isolation of EV

Extracellular vesicles were extracted by ultracentrifugation (Beckman, Miami, FL, USA,Optima L‐100XP). The conditioned medium (CM) was centrifuged at 2000 ***g*** for 15 min after 48 h of cell growth to remove cell debris, and the supernatant was filtered through a 0.22 谬m filter. The filtered supernatant was centrifuged as previously described [15]. For the subsequent experiments, EV pellets were resuspended in protein lysate or fresh medium.

### Characterization of EV

The EV protein content was measured using a BCA protein assay kit (Thermo Fisher Scientific, Waltham, MA, USA). Extracellular vesicles particle size was detected by Transmission Electron Microscope (TEM). Images were obtained using TEM H‐7650. Nanoparticle tracking analysis was used to determine the particle size distribution of EV. Extracellular vesicle platelets were suspended in PBS and detected using zetaview pmx 110 (Particle Metrix, Inning am Ammersee, Germany).

### Endothelial cell secreted media (conditioned media, CM)

Endothelial cells were seeded in 10‐cm plates with complete EBM‐2. When the cells reached approximately 90% confluency, the medium was replaced with serum‐free EBM‐2 and cultured for 36 h. The secreted media were filtered through a 0.22 μm filter after being centrifuged for 10 min at 2000 ***g*** to eliminate cell debris. The fluid was concentrated using an Amicon Ultra‐15 centrifugal filter unit equipped with an Ultracel‐3K membrane (Millipore, Watford, UK). The BCA Protein Assay Kit was used to determine the protein content following centrifugation for 1 h 40 min at 2000 ***g***.

### Protein extraction and western blotting

The cells were collected in Tris‐NaCl‐Triton‐EDTA (TNTE) buffer containing four protease inhibitors (phenylmethylsulfonyl fluoride, leupeptin, pepstatin, and aprotinin), and the total protein concentration was determined using a BCA kit. SDS/PAGE was used to separate equal quantities of samples, and the separated samples were then transferred onto nitrocellulose membranes. (Millipore) After being blocked for 1 h with 5% nonfat milk at room temperature, the membranes were incubated with primary antibodies at 4 °C overnight against Flotillin‐1 (ab41927, Abcam, Cambridge, MA, USA, 1 : 1000), TSG101 (ab83, Abcam, 1 : 1000), MYO1C (sc136544, Santa, Santa Cruz, CA, USA, 1 : 1000), Flag (AE005, ABclonal, Wuhan, China, 1 : 2000), β‐actin (ab8227, Abcam, 1 : 5000), RAB31 (16182‐1‐AP, Proteintech, Chicago, IL, USA, 1 : 500), RAB27B (13412‐1‐AP, Proteintech, 1 : 500), FAS (13098‐1‐AP, Proteintech, 1 : 3000), SDC2 (67088‐1‐Ig, Proteintech, 1 : 1000), TSPAN4 (ab181995, Abcam, 1:500), CD81 (66866‐1‐Ig, Proteintech, 1 : 3000), and CD82 (66803‐1‐Ig, Proteintech, 1 : 3000). Next, the membranes were probed with secondary antibody for 2 h at room temperature. At last, protein signaling was visualized using Pierce™ ECL Western Blotting Substrate (Thermo Fisher Scientific; Amersham) and quantified using the imagej software (NIH, Bethesda, MD, USA).

### Immunoprecipitation

Anti‐FLAG beads (Thermo Fisher Scientific) were washed three times with RIPA buffer prior to exogenous immunoprecipitation (IP). After that, 25 μL of the beads was added and kept at 4 °C with the lysates overnight. Endogenous IP was performed according to the manufacturer's instructions using a Pierce Classic Magnetic IP/Co‐IP Kit (Rockford, IL, USA, #88804).

### Transwell invasion assay

Diluted Matrigel (Corning, Corning, NY, USA) and Transwell inserts with a pore size of 8.0 μm (Corning) were used to conduct the cell invasion assay. A total of 1 × 10^5^ cells per 100 μL were seeded into the upper compartment. In the bottom chamber, 600 μL of the CM or EV suspension was loaded. After 24–48 h of conventional culture, the invading cells were fixed with 4% paraformaldehyde (PFA) and stained with 1% crystal violet. The bottom of the Transwell plate was imaged using an inverted fluorescence microscope (EVOs). Migrated cells were counted using the imagej software. Each experiment consisted of three duplicate wells and was repeated three times.

### Mass spectrometry analysis

We used reversed‐phase nanoliquid chromatography‐tandem mass spectrometry (LC–MS/MS) (Ultimate 3000 coupled to QEx‐active, Thermo Scientific) to analyze the intracellular proteins 17 GhECs and NhECs, as described previously [22].

### Transfections and lentiviral transduction

Cells were seeded on 12‐well plates or 10‐cm petri dishes and transfected with siRNAs or plasmids the next day using INTERFERin® (PolyPlus, IIIkirch, France) or Lipofectamine 3000 (Invitrogen, Carlsbad, CA, USA) according to the manufacturer's protocol. The siRNAs were as follows: siNC sense: 5′‐UUCUCCGAACGUGUCACGUTT‐3′; siRAB31‐1 sense: 5′‐GGAGCUCAAAGUGUGCCUUTT‐3′; siRAB31‐2 sense: 5′‐GCCAUCGUGGUUGAGACAATT‐3′; siSDC2‐1 sense: 5′‐GAAACCACGACGCTGAATATT‐3′; siSDC2‐2 sense: 5′‐GAAACCACGACGCTGAATATT‐3′; siRAB27B‐1 sense: 5′‐CCCAAATTCATCACTACAGTA‐3′; siRAB27B‐2 sense: 5′‐CCAGUCAACAGAGCUUCUUTT‐3′; siFAS‐1 sense: 5′‐GCCAAGAAGGGAAGGAGUATT‐3′; siFAS‐2 sense: 5′‐GCCCAAGUGACUGACAUCATT‐3′; siFAS‐3 sense: 5′‐GGAGUACACAGACAAAGCCTT‐3′; siMYO1C‐1 sense: 5′‐GCUCAAAGAAUCCCAUUAUTT‐3′; siMYO1C‐2 sense: 5′‐CUUGGUACAGAUGAGAUCATT‐3′. Lentiviral particles were purchased from GENECHEM Company (Shanghai, China).

### Statistical analysis

We conducted Gene Ontology analysis by the Database for Annotation, Visualization and Integrated Discovery (david, version 6.8, NIH). Graphing was performed using graphpad prism 6.0 Software (GraphPad Software, San Diego, CA, USA). Student's *t*‐test was used for statistical analysis, and data are presented as mean ± SD. *P*‐value < 0.05 was defined as the cutoff criteria.

## Results

### MYO1C is specifically enriched in GhEC‐EV

**Fig. 1:**
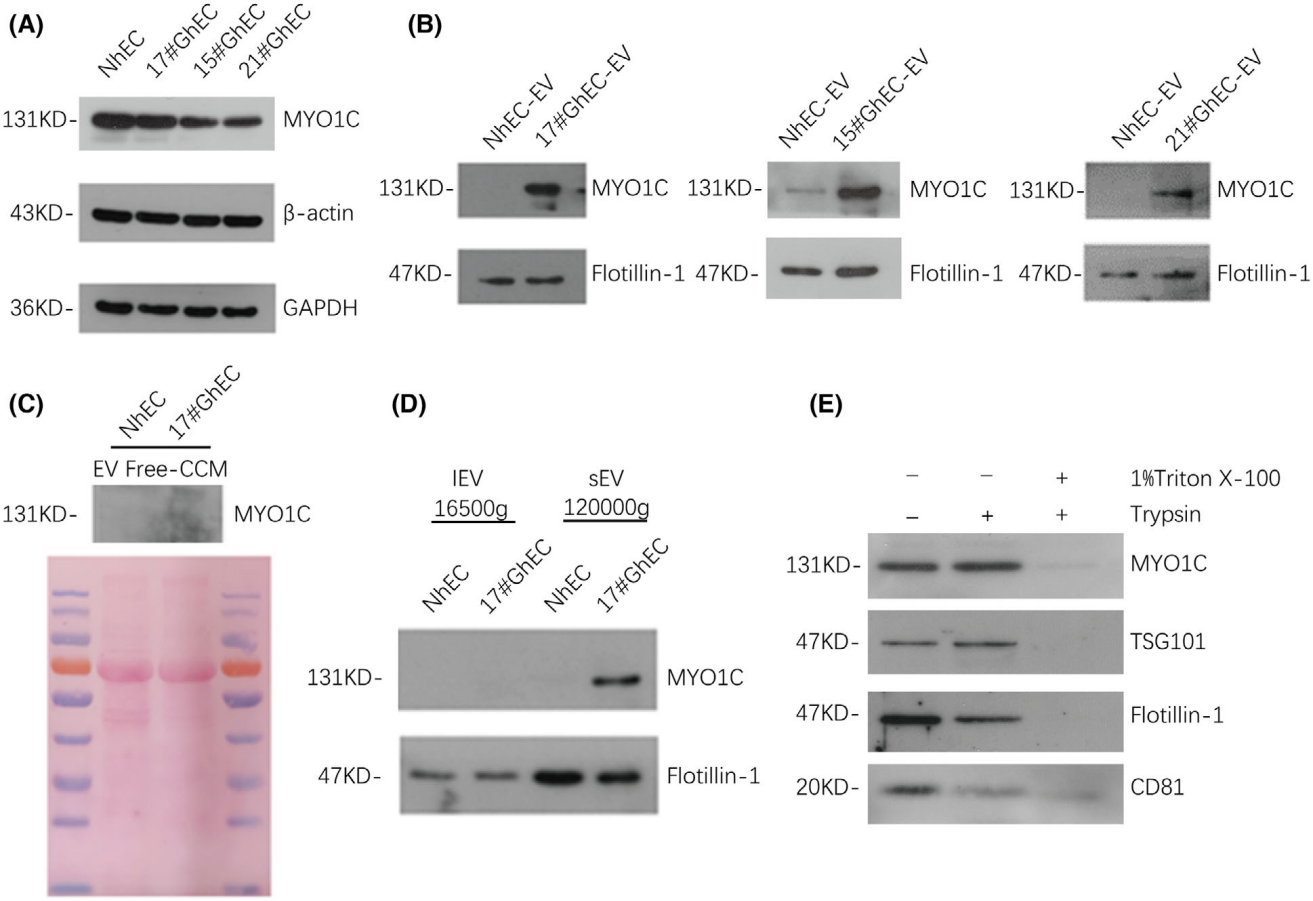
MYO1C is specifically enriched in GhEC‐EV. (A) Western blot analysis of MYO1C expression level in ECs. (B) Western blot analysis of MYO1C expression level in EC‐EV. (C) Western blot analysis of MYO1C expression level in EV‐free CCM (concentrated conditional media). CM was concentrated from the culture supernatant of 2 × 10^7^ GhEC and NhEC. (D) Total protein contained within large extracellular vesicle (lEV) and small extracellular vesicle (sEV) isolated together from the culture supernatants of 3 × 10^7^ GhEC and NhEC. MYO1C is specifically enrichment in sEV of GhEC. (E) Isolated sEV were incubated with trypsin in the presence or absence of 1% of Triton X100. Related protein levels were determined by western blot. The representative images are shown.

In previous research, we have already identified the EC‐derived EV (GhEC and NhEC) by transmission electron microscopy (TEM) and nanoparticle tracking analysis (NTA) and found no differences in their appearance or size [15]. Here, we detected MYO1C protein in cells and EVs of NhECs and GhECs (17#, 21#, 15#) from different glioma tissues. Western blotting showed that MYO1C was significantly more enriched in GhEC‐EVs than in NhEC‐EVs. However, there was no obvious difference in the expression of MYO1C at the cellular level (Fig. 1A,B). To determine the source of MYO1C, we ultracentrifuged the cell culture medium supernatant to remove extracellular vesicles and found that MYO1C could not be detected by western blotting in the components concentrated by ultrafiltration (Fig. 1C). By isolating lEV and sEV from the culture supernatants of GhECs and NhECs, we found that MYO1C was mainly enriched in the sEV of GhECs, suggesting that MYO1C might be exported via sEV (Fig. 1D). To rule out potential contributions from nonvesicular particles, cell culture supernatants were subjected to trypsin‐based degradation assays. We found that incubation with trypsin alone did not significantly alter MYO1C levels. Unlike the membrane proteins flotillin‐1 and CD81, MYO1C is resistant to trypsin, indicating that MYO1C is located inside the sEV, like TSG101. In contrast, MYO1C was almost completely degraded when we treated with a combination of the nonionic surfactants Triton X‐100 and trypsin (Fig. 1E). In summary, we showed that MYO1C was encapsulated into sEV derived from GhECs, thus protecting them from degradation by trypsin.

### Knockdown of RAB31, RAB27B, or FAS suppressed MYO1C enrichment in GhEC‐EV

**Fig. 2:**
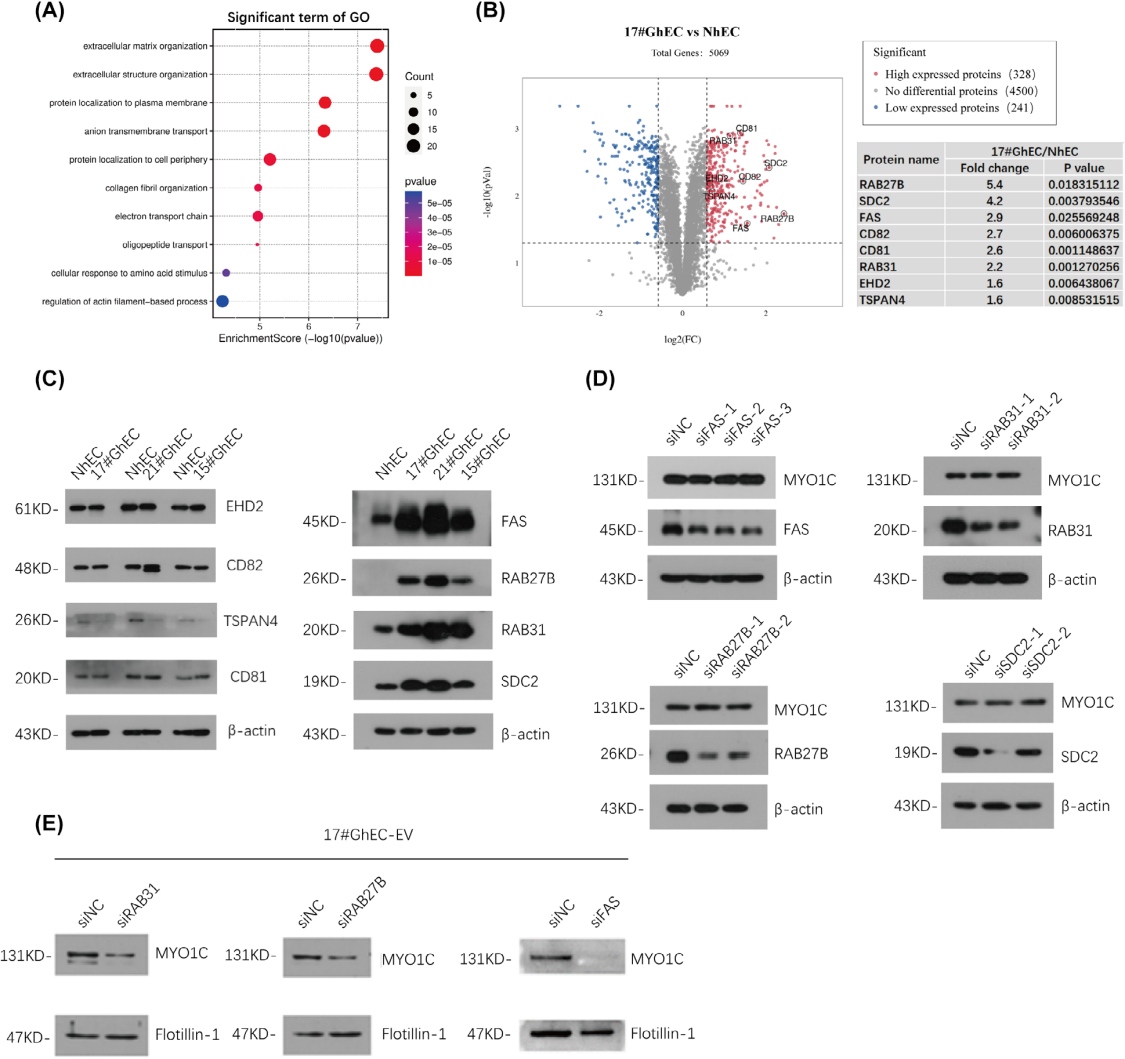
Knockdown of RAB31, RAB27B or FAS reduced MYO1C enrichment in GhEC‐EV. (A, B) Gene Ontology analysis of highly expressed proteins in 17#GhEC. Volcano map of 17#GhEC vs. NhEC mass spectrometry results (FC > 1.5, *P* < 0.05). Eight proteins related to EV formation were chosen. (C) Candidate proteins in GhEC (17#, 21# and 15#) and NhEC were detected by western blotting. (D) After knockdown of FAS, RAB31, RAB27B or SDC2 in GhEC, the cellular MYO1C protein levels were detected. (E) After knockdown of RAB31, RAB27B, or FAS in GhEC, the MYO1C enrichment levels in GhEC‐EV were detected. The representative images are shown.

To screen for specific regulatory factors that mediate MYO1C enrichment in GhEC‐EVs, we used mass spectrometry to analyze the proteins from GhECs and NhECs. Here, we focused on proteins that are highly expressed in GhECs. Gene Ontology enrichment analysis showed that most of them were related to extracellular matrix organization, protein localization, and transport (17#GhEC/NhEC ratio > 1.5) (Fig. 2A). Subsequently, combining with previous reports, eight proteins were screened from these 328 highly expressed proteins that may be involved in extracellular vesicle formation (Fig. 2B and Table S1). To verify the reliability of mass spectrometry, we measured the expression levels of RAB31, RAB27B, FAS, CD81, CD82, TSPAN4, EHD2, and SDC2 by western blotting. The results showed that RAB31, RAB27B, FAS, and SDC2 were expressed at higher levels in GhECs than in NhECs. However, CD81, CD82, EHD2, and TSPAN4 levels were not significantly different between GhECs and NhECs (Fig. 2C). To further confirm the contribution of these proteins in the export of MYO1C, we first interfered with their expression using siRNA‐mediated knockdown in GhECs. We found that inhibiting the expression of these proteins did not significantly alter the cellular levels of MYO1C (Fig. 2D). However, with the exception of SDC2 silencing (Fig. S1), inhibition of RAB31, RAB27B and FAS protein expression strongly interfered with MYO1C export compared to that in the control conditions (Fig. 2E).

### RAB31 mediates the MYO1C enrichment in GhEC‐EV

**Fig. 3:**
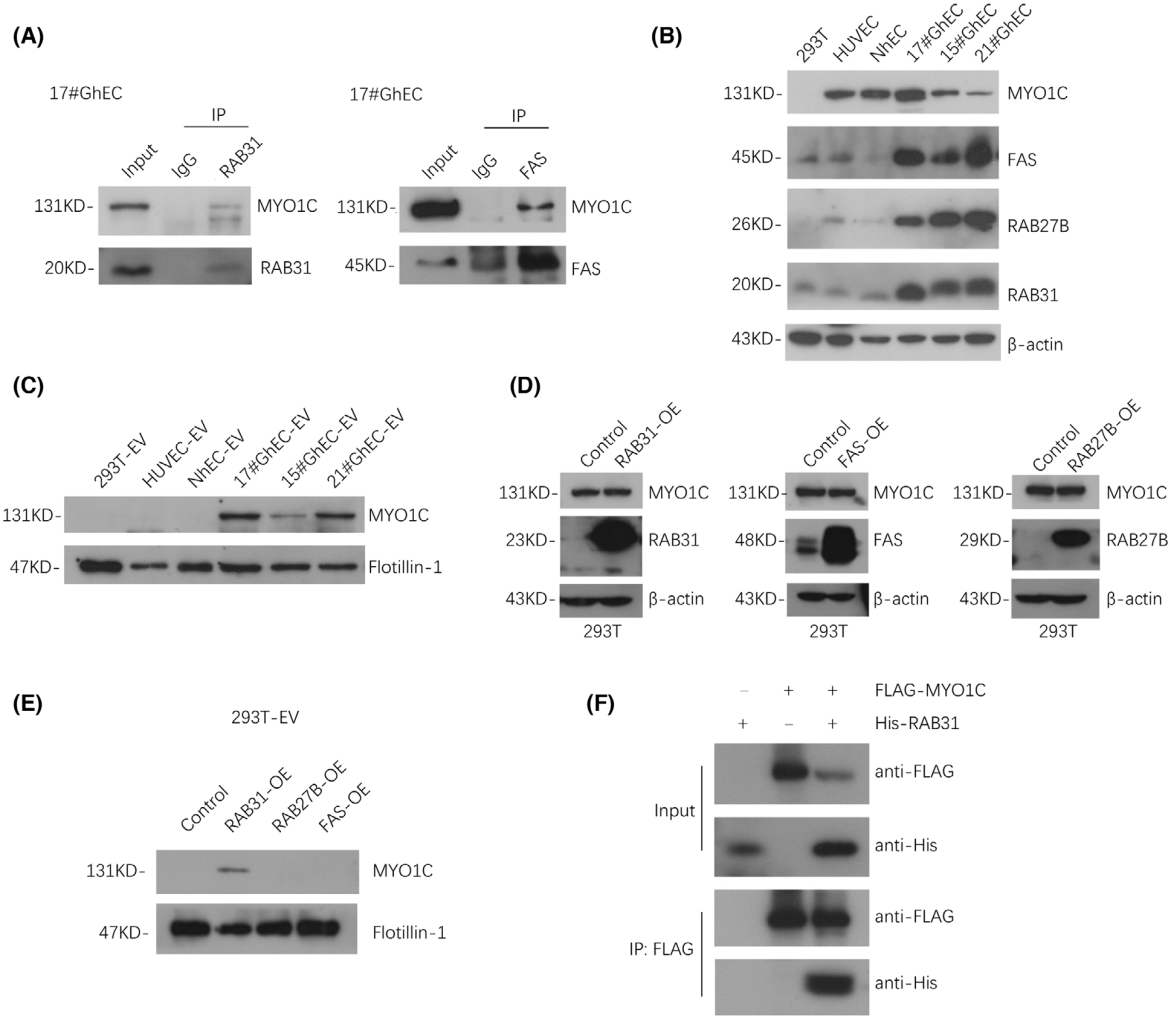
RAB31 may mediate the enrichment of MYO1C in GhEC‐EV. (A) Immunoprecipitation of RAB31 and FAS protein in 17#GhEC. (B) Western blot analysis MYO1C, RAB31, RAB27B, and FAS expression level in 293T cells, HUVEC, NhEC, and GhECs(17#, 15#, 21#). (C) Western blot analysis of the enrichment of MYO1C in 293T‐EV, HUVEC‐EV, NhEC‐EV, and GhEC‐EV. (D, E) After overexpression of RAB31, RAB27B, or FAS in 293T cells, the MYO1C level increased in 293T cells and 293T‐EV was detected. (F) Exogenous immunoprecipitation in 293T cells was detected. The representative images are shown.

We further investigated the relationship between RAB31, RAB27B, and FAS and MYO1C export. RAB31 and FAS could bind to MYO1C at endogenous levels in immunoprecipitation experiments in GhECs, whereas no significant interaction between RAB27B and MYO1C was detected (Fig. 3A and Fig. S2). Based on previous reports, we hypothesized that these three proteins are involved in different MYO1C export processes. Subsequently, we found that the expression levels of these three proteins in 293T cells were similar to those in NhECs and human umbilical vein endothelial cells (HUVEC) (Fig. 3B), and the enrichment of MYO1C in 293T‐EV was as low as that in NhEC‐EVs and HUVEC‐EVs (Fig. 3C). Therefore, we hypothesized that 293T cells have an EV secretion pattern similar to that of NhECs. To further explore the mechanism by which these proteins regulate MYO1C export, we overexpressed RAB31, RAB27B, and FAS in 293T cells and found that MYO1C was enriched in EVs only when RAB31 was overexpressed, without significant changes at the cellular level (Fig. 3D,E). Exogenous immunoprecipitation in 293T cells showed that MYO1C could bind to RAB31 (Fig. 3F). In summary, RAB31 may play a key role in the enrichment of MYO1C in EVs.

### Knockdown of RAB31 reduces the invasion of glioma cells mediated by GhEC‐EV

**Fig. 4:**
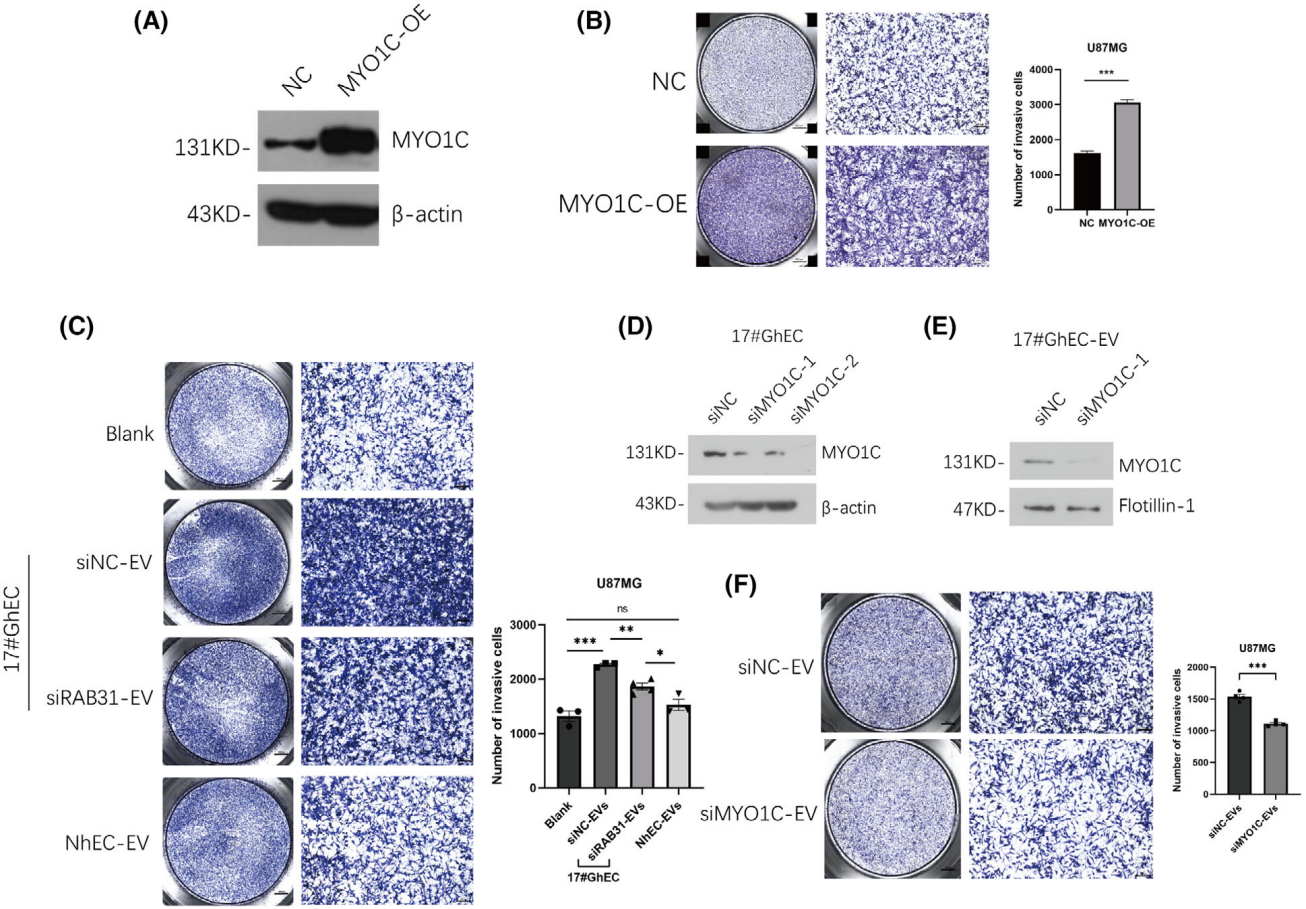
Knockdown of RAB31 reduces the invasion of glioma cells mediated by GhEC‐EV. (A) MYO1C was overexpressed in U87MG cells by lentiviral transduction and expression levels were determined by western blotting. (B) Transwell invasion assays evaluated the effect of MYO1C overexpression in U87MG cells (*n* = 3). (C) The effect of 17#GhEC‐siRAB31‐EVs, 17#GhEC‐siNC‐EVs or NhEC‐EVs on the invasion ability of U87MG cells (EVs quantity: 10 μg EVs per well). Data are presented as the mean ± SEM (*P < 0.05, **P < 0.01, ***P < 0.001, Student's *t*‐test). Scale bar: 800 μm, left; 100 μm, right. (D) Analysis the siRNA‐mediated knockdown of MYO1C in 17#GhEC. (E) Analysis the MYO1C content in 17#GhEC‐siMYO1C‐EVs. (F) Transwell invasion assays showed change in the invasive cells number of U87M treated with siNC‐EVs or siMYO1C‐EVs from 17#GhEC (EVs quantity: 10 μg EVs per well).

Based on these findings, we explored the significance of this extracellular vesicle regulatory mechanism in glioma progression. We overexpressed MYO1C in glioma cells (GCs) and glioma stem cells (GSCs) and found that it promoted their invasive ability. The different effects on U87MG and GSC2 cells in terms of cell proliferation capacity are also shown (Fig. 4A,B and Fig. S3A–C), which might be due to their different relative abundance of MYO1C. To evaluate the effect of MYO1C‐rich EVs mediated by RAB31 on glioma progression, U87MG cells and GSC2 were treated with EVs from control GhECs (siNC), RAB31 knockdown GhEC (siRAB31), or NhECs. Compared with GhEC‐derived siNC‐EVs and NhEC‐derived EV, GhEC‐derived siRAB31‐EV significantly decreased cell invasion (Fig. 4C and Fig. S3D). Subsequently, to further confirm the function of Rab31‐mediated MYO1C cargo, we knocked down MYO1C in 17#GhEC and found that MYO1C was also decreased in EVs (Fig. 4D,E). Then, we used these EVs to treat glioma cells and found that the invasion ability of glioma cells in the siMYO1C‐EVs treatment group was weaker than that in the siNC‐EVs treatment group (Fig. 4F). The results showed that siMYO1C‐EVs had similar biological effects to siRAB31‐EVs, which explained the function of MYO1C cargo mediated by RAB31 to a certain extent. In addition, the GhEC‐EV revealed no significant differences in size or appearance of after RAB31 knockdown (Fig. S4A,B). In summary, our data illustrate that the knockdown of RAB31 can reduce the enrichment of MYO1C in extracellular vesicles, thereby attenuating the promotion of glioma cell invasion by GhEC‐EVs.

## Discussion

In this study, we focused on the specific EVs secretion mechanism of GhECs, which differs from that of NhECs. In previous studies, we found no significant difference in the expression of MYO1C at the cellular level between GhECs and NhECs; however, MYO1C was only enriched in GhEC‐EVs. We isolated EV of endothelial cells and conducted particle size analysis, morphological detection, and western blotting for EV marker proteins [15, 22]. We further excluded the interference of free components and nonmembrane structures in the cell culture medium supernatants, and EV pellets were subjected to trypsin degradation assays. Our results show that MYO1C in GhECs was loaded into sEV and secreted.

Intracellular cargo is loaded into intraluminal vesicles (ILVs) by the inward budding of multivesicular endosomes (MVEs) and is exported by the fusion of MVEs with the cell membrane [11, 14]. To explore the mechanism underlying the different secretion patterns of GhECs and NhECs, we analyzed the mass spectrometry data of endothelial cell proteins and screened eight proteins that may be involved in extracellular vesicle secretion. According to previous studies, RAB27B controls MVEs docking at the plasma membrane to regulate exosome secretion in HeLa cells [24]. Another RAB family member, RAB31, can combine with flotillin proteins in lipid raft microdomains to promote the formation of ILVs by MVEs and prevent the fusion of MVE with lysosomes by inhibiting RAB7 activity [25]. In addition, studies have shown that as a membrane protein, FAS/CD95 mediates membrane fusion to release small extracellular vesicles (sEV) from MSCs [26]. Our results showed that knockdown of these proteins may inhibit the secretion of MYO1C, and the immunoprecipitation results showed that these three proteins may play a role in different stages of sEV secretion.

Given that the protein expression profile of 293T cells was similar to those of NhECs and HUVEC, we hypothesized that the EVs secretion mode of 293T cells may be similar to that of NhECs. We used 293T cells as a model to further explore the proteins that play key roles in MYO1C secretion. We found that only when RAB31 was overexpressed, the degree of MYO1C in 293T‐EV increased. Combined with previous research reports on the function of these three proteins, we speculate that RAB27B and FAS may play a role in the fusion of MVEs with cell membranes and that RAB31 may mediate inward budding on MVEs to load MYO1C into ILVs. From another perspective, the low enrichment of MYO1C in NhEC‐EVs may be due to the lack of a pathway to load MYO1C or an ILVs export mechanism that leads to MVEs targeting lysosomes for degradation.

MYO1C is involved in several biological processes related to membrane transport and motility. Studies have shown that classical endosomal and autophagic pathways maintain cell health by selectively degrading cargo through fusion with lysosomes [27, 28]. The loss of functional MYO1C results in a disordered distribution of lipid components in the cell, thereby disrupting protein degradation via the autophagy‐lysosomal pathway. MYO1C has been reported to mediate vesicular trafficking in cells; here, we focused on its role as a vesicular cargo [29]. It is also worth noting that myosin 1C isoform A is a novel candidate diagnostic marker for prostate cancer [30]. Our results demonstrated that MYO1C overexpression can promote the aggressiveness of U87MG cells, while in GSC2, it appeared to promote the cell proliferation capacity; this differential effect may be due to the difference in cellular expression levels of MYO1C protein or stem cell characteristics. In addition, the knockdown of RAB31 significantly attenuated the promotion of glioma cell invasion by GhEC‐EVs.

This study aimed to explore the mechanisms underlying MYO1C enrichment in GhEC‐EVs. Tumor endothelial cells have genetic characteristics and biological phenotypes that are different from those of normal endothelial cells, which enhance the susceptibility of tumor vessels to antiangiogenic therapeutic strategies [31], indicating that vascular heterogeneity may be a key target for tumor vascular therapy. Here, we found the expression of RAB31 in glioma vascular endothelial cells and normal cerebral vascular endothelial cells is heterogeneous, and the effect of RAB31 on glioma angiogenesis needs to be further studied.

## Conflict of interest

The authors declare no conflict of interest.

### Peer review

The peer review history for this article is available at https://www.webofscience.com/api/gateway/wos/peer‐review/10.1002/2211‐5463.13736.

## Author contributions

WH conceived the project and designed the experiments. JS performed the experiments and wrote the manuscript. YW, LW, BQiu, and AY assisted with data analysis. XP, BQia, and ZW critically read the manuscript. All the authors have read and approved the final manuscript before submission.

## Supporting information


**Fig. S1.** Western blot analysis of MYO1C protein level in 17#GhEC‐EV when SDC2 was knocked down.
**Fig. S2.** Endogenous immunoprecipitation of RAB27B in 17#GhEC.
**Fig. S3.** MYO1C was overexpressed in GSC2 by lentiviral transduction and expression levels were determined by western blot.
**Fig. S4.** Nanoparticle‐tracking analysis (NTA) of siNC‐EV and siRAB31‐EV of 17#GhEC.

**Table S1.** 328 highly expressed proteins in GhEC (GhEC vs. NhEC).

## Data Availability

The data that support the findings of this study are available in the Supporting Information of this article.
