# Supplementary material for: RAB31 in glioma‐derived endothelial cells promotes glioma cell invasion via extracellular vesicle‐mediated enrichment of MYO1C
Source: FEBS Open Bio. 2023 Nov 20;14(1):138–47. doi: 10.1002/2211-5463.13736 (PMC10761932; doi:10.1002/2211-5463.13736)
Supplement: Supplementary file 1 — Fig. S1. Western blot analysis of MYO1C protein level in 17#GhEC‐EV when SDC2 was knocked down. Fig. S2. Endogenous immunoprecipitation of RAB27B in 17#GhEC. Fig. S3. MYO1C was overexpressed in GSC2 by lentiviral transduction and expression levels were determined by western blot. Fig. S4. Nanoparticle‐tracking analysis (NTA) of siNC‐EV and siRAB31‐EV of 17#GhEC. [file FEB4-14-138-s001.docx]

Supplementary data

The following are the Supplementary data to this article:


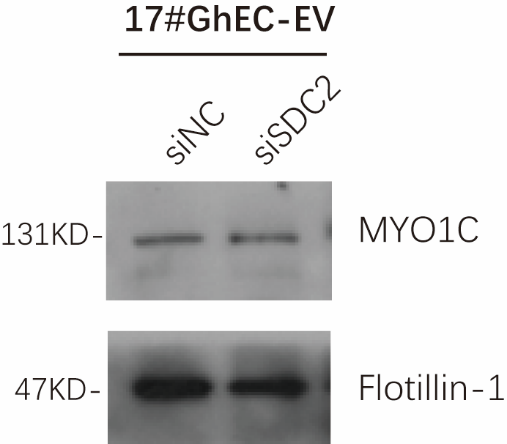


**Supplementary Fig.1** Western blot analysis of MYO1C protein level in 17#GhEC-EV when SDC2 was knocked down. The representative image was showed.


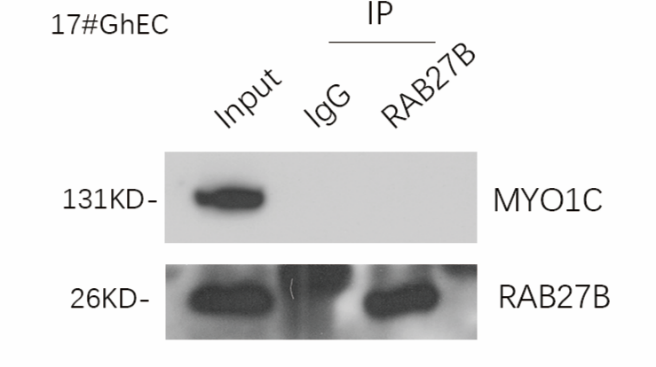


**Supplementary Fig.2** Endogenous immunoprecipitation of RAB27B in 17#GhEC. Western blotting was performed to analyze the interaction between RAB27B and MYO1C.

**
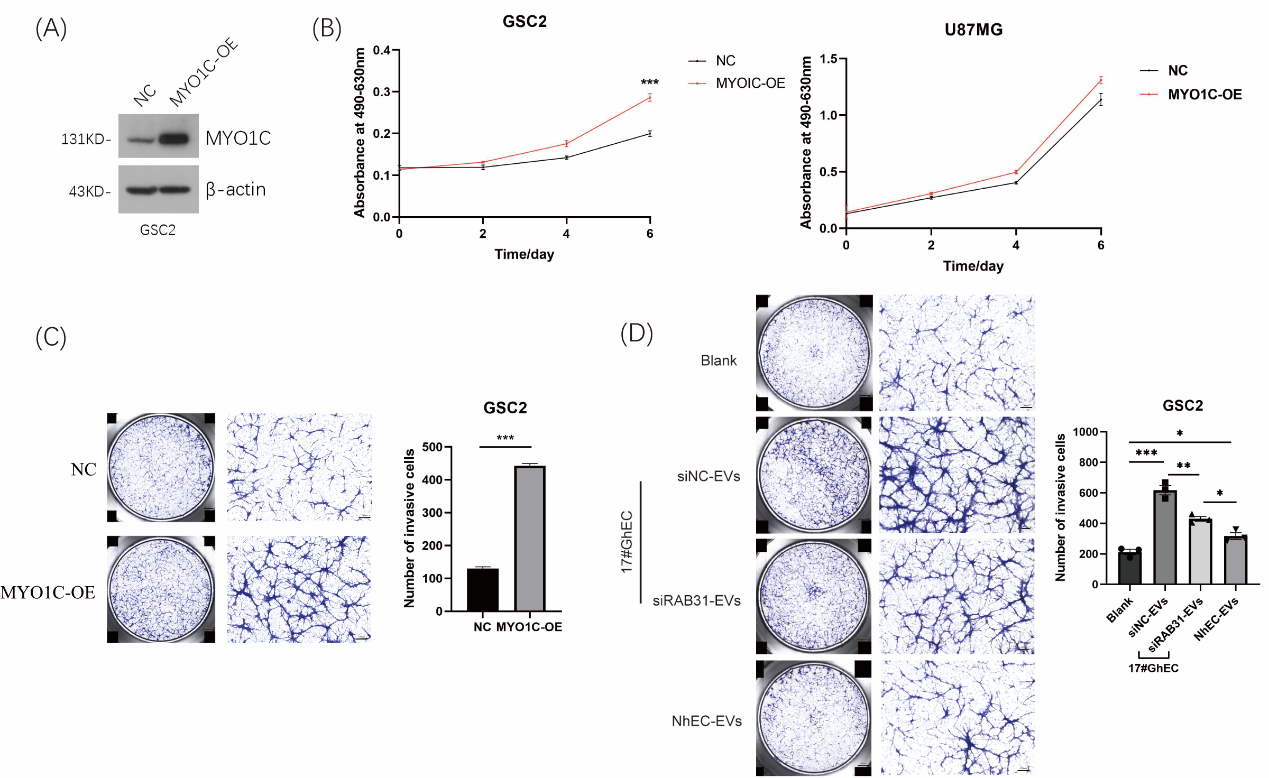
**

**Supplementary Fig.3** (A) MYO1C was overexpressed in GSC2 by lentiviral transduction and expression levels were determined by western blot. (B) GSC2 and U87MG were seeded in a 96-well plate (5.0 × 10^3^ cells/well) and subjected to MTS assay. (C) Transwell invasion assay evaluated the effect of MYO1C overexpression in GSC2 (n = 3). The representative images were showed. Data are presented as the mean ± SEM (*P < 0.05, **P < 0.01, ***P < 0.001, Student’s t-test). Scale bar: 800 μm, left; 100 μm, right. (D) The effect of 17#GhEC-siRAB31-EVs, 17#GhEC-siNC-EVs or NhEC-EVs on the invasion ability of GSC2 (EVs quantity: 10μg EVs/well). Data are presented as the mean ± SEM (*P < 0.05, **P < 0.01, ***P < 0.001, Student’s t-test) Scale bar: 800 μm, left; 100 μm, right.


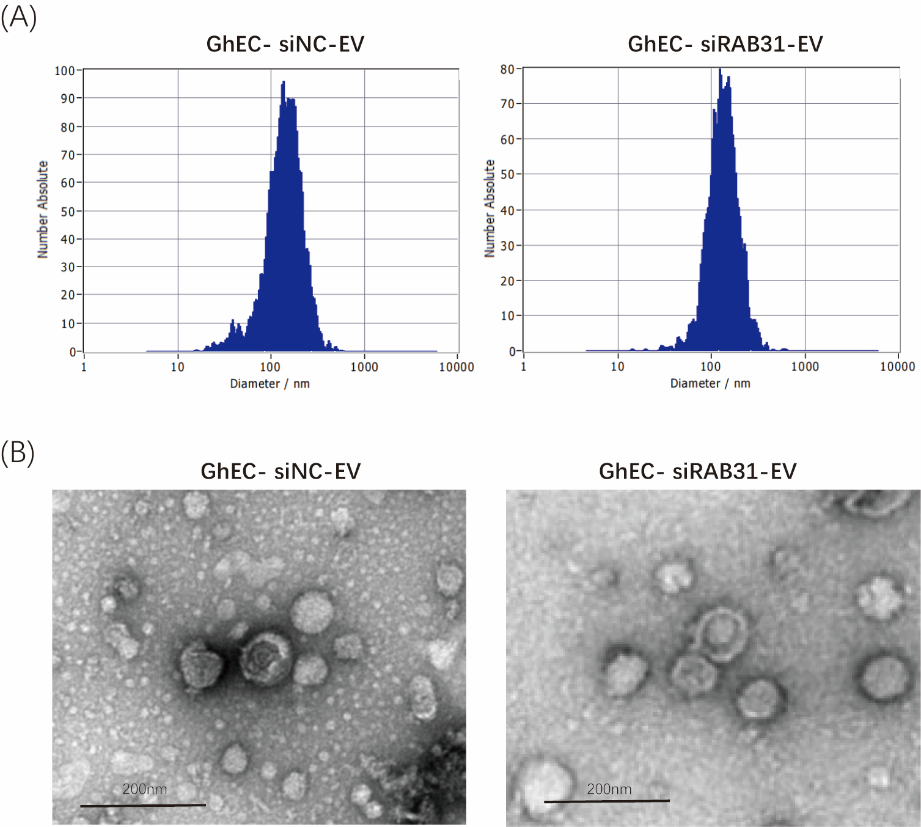


**Supplementary Fig.4** (A)Nanoparticle-tracking analysis (NTA) of siNC-EV and siRAB31-EV of 17#GhEC. EVs (1μg) were dissolved in 1ml PBS. (B) Transmission Electron Microscope (TEM) images of siNC-EV and siRAB31-EV of 17#GhEC. Scale bars: 200 nm.
